# Discovery of a small-molecule protein kinase Cδ-selective activator with promising application in colon cancer therapy

**DOI:** 10.1038/s41419-017-0154-9

**Published:** 2018-01-18

**Authors:** Cláudia Bessa, Joana Soares, Liliana Raimundo, Joana B. Loureiro, Célia Gomes, Flávio Reis, Miguel L. Soares, Daniel Santos, Chetna Dureja, Saumya R. Chaudhuri, Cynthia Lopez-Haber, Marcelo G. Kazanietz, Jorge Gonçalves, Maria F. Simões, Patrícia Rijo, Lucília Saraiva

**Affiliations:** 1 UCIBIO/REQUIMTE, Laboratório de Microbiologia, Departamento de Ciências Biológicas, Faculdade de Farmácia, Universidade do Porto, Porto, Portugal; 2 Laboratory of Pharmacology and Experimental Therapeutics, Institute for Biomedical Imaging and Life Sciences (IBILI), Faculty of Medicine, & CNC.IBILI Research Consortium, University of Coimbra, Coimbra, Portugal; 3 Laboratório de Apoio à Investigação em Medicina Molecular, Departamento de Biomedicina, Faculdade de Medicina da Universidade do Porto, Porto, Portugal; 4 REQUIMTE, Faculdade de Ciências da Universidade do Porto, Porto, Portugal; 5 CSIR-Institute of Microbial Technology, Sector 39A, Chandigarh, India; 6 Department of Systems Pharmacology and Translational Therapeutics, Perelman School of Medicine, University of Pennsylvania, Philadelphia, USA; 7 Laboratório de Farmacologia, Departamento de Ciências do Medicamento, Faculdade de Farmácia, Universidade do Porto, Porto, Portugal; 8 CBIOS-Centro de Investigação em Biociências e Tecnologias da Saúde, Universidade Lusófona, Lisboa, Portugal; 9 iMed.ULisboa, Instituto de Investigação do Medicamento, Faculdade de Farmácia da Universidade de Lisboa, Lisboa, Portugal

## Abstract

Protein kinase C (PKC) isozymes play major roles in human diseases, including cancer. Yet, the poor understanding of isozymes-specific functions and the limited availability of selective pharmacological modulators of PKC isozymes have limited the clinical translation of PKC-targeting agents. Here, we report the first small-molecule PKCδ-selective activator, the 7*α*-acetoxy-6*β*-benzoyloxy-12-*O*-benzoylroyleanone (Roy-Bz), which binds to the PKCδ-C1-domain. Roy-Bz potently inhibited the proliferation of colon cancer cells by inducing a PKCδ-dependent mitochondrial apoptotic pathway involving caspase-3 activation. In HCT116 colon cancer cells, Roy-Bz specifically triggered the translocation of PKCδ but not other phorbol ester responsive PKCs. Roy-Bz caused a marked inhibition in migration of HCT116 cells in a PKCδ-dependent manner. Additionally, the impairment of colonosphere growth and formation, associated with depletion of stemness markers, indicate that Roy-Bz also targets drug-resistant cancer stem cells, preventing tumor dissemination and recurrence. Notably, in xenograft mouse models, Roy-Bz showed a PKCδ-dependent antitumor effect, through anti-proliferative, pro-apoptotic, and anti-angiogenic activities. Besides, Roy-Bz was non-genotoxic, and in vivo it had no apparent toxic side effects. Collectively, our findings reveal a novel promising anticancer drug candidate. Most importantly, Roy-Bz opens the way to a new era on PKC biology and pharmacology, contributing to the potential redefinition of the structural requirements of isozyme-selective agents, and to the re-establishment of PKC isozymes as feasible therapeutic targets in human diseases.

## Introduction

Protein kinase C (PKC) is a family of serine-threonine kinases grouped based on their distinct regulation into “conventional” (cPKCs α, βΙ, βΙΙ, γ), “novel” (nPKCs δ, ε, η, θ), and “atypical” (aPKCs ι/λ, ζ). PKC isozymes share a conserved N-terminal regulatory region comprising the C1-domain and C2-domain, and a C-terminal catalytic region responsible for ATP binding and phosphotransferase activity. The C1-domain is the phorbol ester and diacylglycerol (DAG) binding site in cPKCs and nPKCs, where it is duplicated in tandem (C1a and C1b). The C2-domain in cPKCs binds calcium, whereas in nPKCs it is primarily a calcium-unresponsive phospho-tyrosine binding motif. On the other hand, aPKCs bind neither phorbol ester/DAG nor calcium^1–3^. PKC isozymes are involved in the activation of multiple signaling pathways, and hence they are recognized therapeutic targets for several human diseases. Their wide-ranging effects in crucial processes of tumorigenesis and metastatic dissemination justify the efforts to develop PKC-targeted drugs for cancer treatment. Yet, the pharmacological modulation of PKCs in anticancer therapy has proved generally ineffective in clinical trials. This can be explained by the complex biological functions regulated by PKC isozymes, both redundant and opposite, and their significant expression heterogeneity in different cancer types. Unfortunately, there is a very limited availability of isozyme-selective PKC modulators, which represent a major limitation for achieving therapeutic success. As such, the generation of isozyme-selective PKC modulators has been of high priority. However, this has been a major challenge from a pharmacological standpoint, as PKCs are highly related among them, as well as to other structurally related kinases^1–3^.

Whereas PKCs have been generally viewed as oncogenic kinases, several laboratories have established that PKC activity is often lost in cancer due to loss-of-function mutations, thus supporting a potential function of PKC isozymes as tumor suppressors rather than tumor promoters^3^. Moreover, specific PKC isozymes induce anti-proliferative or pro-apoptotic effects in cancer cells upon activation^4–7^. This led researchers to believe that cancer therapies should be focused on restoring rather than inhibiting their activity, and may justify the ineffectiveness of more than three decades of clinical trials using non-selective PKC inhibitors. Therefore, the selective PKC activation may be a valuable therapeutic strategy in cancer treatment^3–7^.

For long, PKC signaling has been closely associated with intestinal carcinogenesis^5,8,9^, one of the most prevalent cancers and a leading cause of cancer mortality worldwide^10^. Particularly, clinical data have revealed reduced protein levels of PKCδ in colon cancer tissues compared to normal tissues^11^. In fact, several studies corroborated the notion that PKCδ behaves as tumor suppressor in colon cancer^11–15^. Therefore, besides their potential application in other cancer types, it is highly conceivable that PKCδ-selective activators may be therapeutically beneficial for patients with colon cancer. Here, we identified the compound 7α-acetoxy-6β-benzoyloxy-12-O-benzoylroyleanone (Roy-Bz) as a PKCδ-selective activator with promising targeted anticancer activity in colon cancer. Importantly, Roy-Bz may also represent a valuable therapeutic alternative in other human pathologies involving an impairment of PKCδ-signaling pathway.

## Results

### Roy-Bz is a selective activator of PKCδ that binds to the C1-domain

**Fig. 1:**
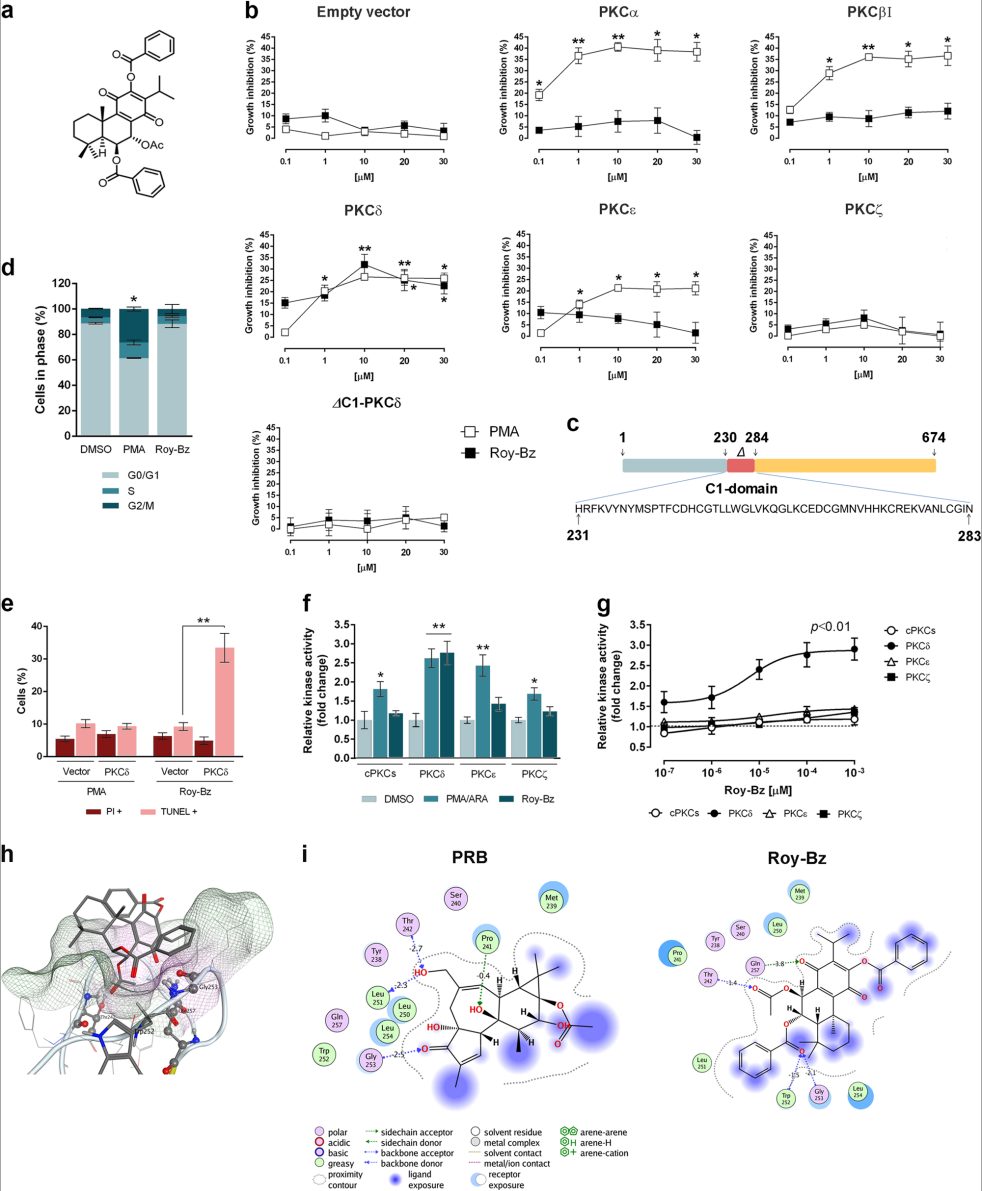
Roy-Bz selectively activates PKCδ through binding to the C1-domain **a** Chemical structure of Roy-Bz. **b** Dose–response curves for the inhibitory effect of PMA and Roy-Bz on the growth of yeast expressing mammalian PKC isozymes and control yeast (empty vector), for 42 h treatment; growth of yeast incubated with vehicle was set as 100%. **c** Schematic representation of deletion of C1-domain at amino acids 231–283 in the *PRKCD* gene. **d**, **e** Effect of 10 μM PMA and Roy-Bz on (**d**) cell cycle progression and (**e**) DNA fragmentation (TUNEL+)/loss of plasma membrane integrity (PI+) of PKCδ-expressing yeast (and control yeast in (**e**)), for 42 h treatment. **f**,** g** In vitro kinase assay with recombinant PKCs; **f** increase of PKC activity by 10^−4^ μM PMA/ARA and 10^−4^ μM Roy-Bz; **g** dose–response curves for the increase of PKC activity by Roy-Bz; kinase activity of endogenous PKC activator phosphatidylserine was set as 1; two-way analysis of variance (ANOVA) (*p* < 0.01). **h**,** i** Molecular docking studies for Roy-Bz interaction with the C1-domain of PKCδ. **h** Top ranked docking pose of Roy-Bz with Gly253, Thr242, and Gln257 highlighted through a CPK representation. The hydrogen bonds between Roy-Bz and these residues are also displayed through a dotted blue line. The interaction between the alpha-hydrogen of Trp252 and the carbonyl (CH•••O) is also represented and the residue is highlighted in licorice. All residues within 4.5 Å of Roy-Bz are represented and the molecular surface of the pocket is displayed through a mesh that changes from green in lipophilic regions to pink (hydrophilic). **i** Scheme of PRB and Roy-Bz interactions. The pocket proximity contour is represented by gray dotted lines, the ligand exposure is depicted in violet and the receptor exposure in blue. Direct interactions are represented by a dotted or green line along with the interaction energy (kcal/mol) estimated by MOE. In **b**, **d**–**f**, data are mean ± SEM of five independent experiments; values significantly different from control yeast **b**, **e** or vehicle **d**, **f**: *p < 0.05, **p < 0.01, unpaired Student’s *t*-test

In order to identify novel PKC activator agents, several natural diterpenoids isolated from plant species belonging to the Lamiaceae family and semi-synthetic derivatives were tested using a previously developed yeast PKC screening assay^16^. In this assay, PKC activators induce a significant growth inhibition in mammalian PKC-expressing yeast, which is proportional to the degree of PKC activation, having no effect on control yeast (empty vector)^16^. Using this approach, it was observed that whereas 0.1–30 μM phorbol 12-myristate 13-acetate (PMA; an established activator of cPKCs and nPKCs) inhibited the growth of yeast-expressing cPKCs (α and βI) and nPKCs (δ and ε), 0.1–30 μM of Roy-Bz (Fig. 1a) only inhibited the growth of PKCδ-expressing yeast without significant effects on yeast expressing other PKCs (Fig. 1b). Interestingly, in PKCδ-expressing yeast, while PMA-induced growth inhibition was associated with G2/M-phase cell cycle arrest, Roy-Bz-induced growth inhibition was mediated by apoptotic cell death, as demonstrated by the increase in DNA fragmentation with preservation of plasma membrane integrity (Fig. 1d, e). Additionally, Roy-Bz-induced growth inhibition was completely abolished in yeast expressing a PKCδ in which the C1-domain has been deleted (*Δ*C1-PKCδ; Fig. 1c) (Fig. 1b). In fact, a similar result was obtained with PMA, which is known to bind PKCs at the C1-domain (Fig. 1b). These results indicate that Roy-Bz is a PKCδ-selective activator and, like PMA, it should have the C1-domain as the predicted binding site.

To confirm the PKC isozyme selectivity observed in the yeast assay, we next performed an in vitro kinase assay using recombinant human cPKCs (a mix of PKCα, β, and γ), nPKCδ, nPKCε, and aPKCζ. All recombinant PKCs were effectively activated at 10^−4^ µM PMA/arachidonic acid (ARA) (positive controls: PMA for cPKCs and nPKCs; ARA for aPKCζ) (Fig. 1f). Remarkably, 10^−7^–10^−3^ μM Roy-Bz only increased the activity of PKCδ (Fig. 1g). Roy-Bz activated PKCδ with an EC_50_ (concentration required to induce 50% effect) value of 58.8 ± 3.6 nM (*n* = 5), similar to that of PMA (61.9 ± 4.3 nM, *n* = 5). Thus, the in vitro kinase assays, in accordance with the yeast assays, revealed a remarkable selectivity of Roy-Bz for PKCδ. Besides, these results show that Roy-Bz is as effective as PMA to activate PKCδ.

Next, the potential binding mode of Roy-Bz to PKCδ was explored by molecular docking studies (Fig. 1h, i). Like 13-acetylphorbol (PRB; positive control), which establishes direct strong hydrogen bond interactions with Gly253 and Thr242 (hydrogen bond donors), and Leu251 (hydrogen bond acceptor) in the C1-domain of PKCδ, Roy-Bz also interacted with Gly253 and Thr242, but exchanged Leu251 with Gln257, all acting as hydrogen donors to carbonyl oxygen functional groups. It is interesting to note that like in the case of PRB, these interactions also stabilized the molecule from both sides of the cleft. The estimated autodock free energy of binding for PRB was −7.54 kcal/mol and for Roy-Bz was −7.87 kcal/mol, and a clustering analysis of the molecular docking results showed other three poses similar to the top-ranked (the most populated one; in a total of 10 poses and 6 different clusters). These predicted binding models support the Roy-Bz binding to the PKCδ-C1-domain.

**Fig. 2:**
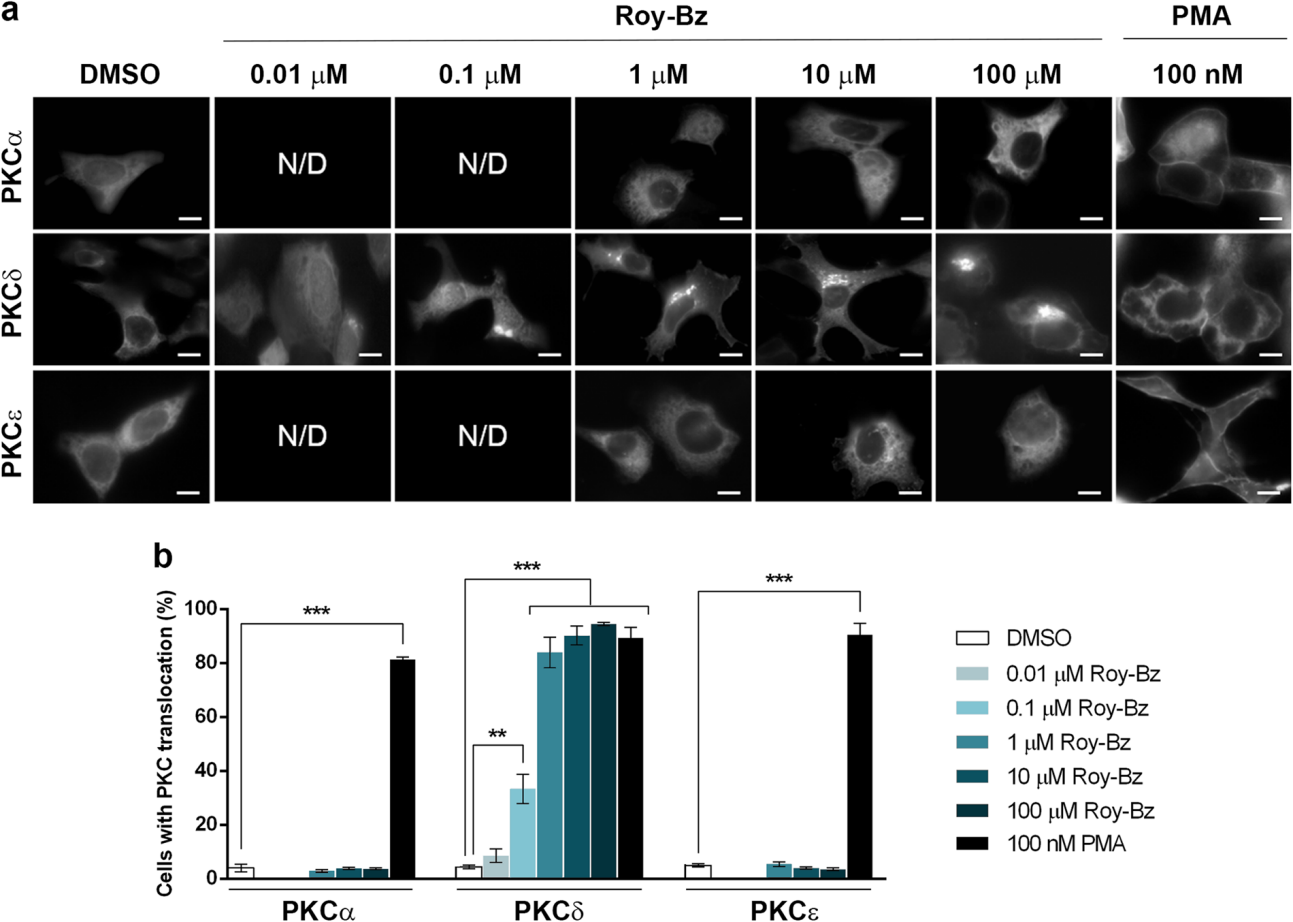
Roy-Bz selectively translocates PKCδ to the perinuclear region in human tumor cells HCT116 cells transfected with pEGFP-N1-PKCα, pEGFP-N1-PKCδ, or pEGFP-N1-PKCε were treated with PMA, Roy-Bz, or vehicle for 1 h; PKC localization was determined by confocal microscopy. **a** Images are representative of three independent experiments; scale bar = 10 µm and magnification = ×100. **b** Quantification of cells with PKC translocation; data are mean ± SEM of three independent experiments; values significantly different are indicated (***p < 0.001), unpaired Student’s *t*-test

The translocation of PKC from the cytosol to membranes, either plasma membrane or internal membranes, is a well-known hallmark of PKC activation^1,17^. As such, we evaluated the impact of Roy-Bz on the translocation of PKCα, PKCδ, and PKCε in human HCT116 colon cancer cells using GFP-fused PKCs (Fig. 2a, b). As previously reported in LNCaP prostate cancer cells^18^, also in HCT116 cells the treatment with PMA triggered the subcellular redistribution of the three PKC isozymes. There was a clear translocation to the plasma membrane for PKCα, PKCδ and PKCε, and as reported in other models^18^, PKCδ also translocated to a perinuclear compartment. When we assessed the ability of Roy-Bz to translocate PKCs, there were obvious differences with regard to PMA. Indeed, Roy-Bz was unable to translocate PKCα or PKCε, while it selectively translocated PKCδ to the perinuclear region (Fig. 2a, b). A dose-dependent analysis of PKCδ translocation in HCT116 cells revealed an EC_50_ value of 0.19 ± 0.07 μM (*n* = 3).

Altogether, these results strongly indicate that Roy-Bz is a selective PKCδ activator, acting at the C1-domain.

### Roy-Bz inhibits the proliferation of colon cancer cells

**Fig. 3:**
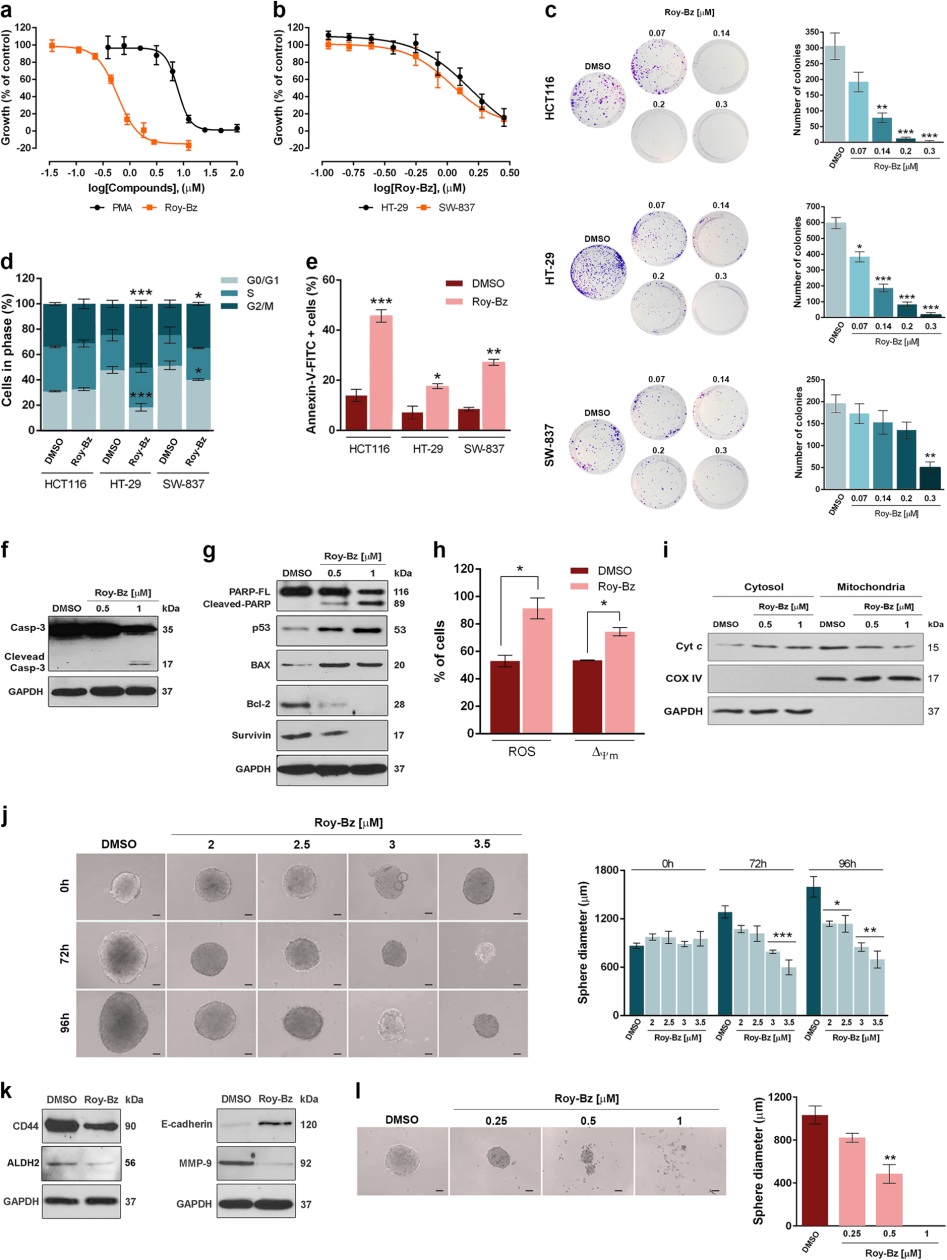
Roy-Bz inhibits the growth of colon cancer cells **a**, **b** Dose–response curves for the growth of (**a**) HCT116, (**b**) HT-29 and SW-837 cells treated with 0.11–10 μM Roy-Bz or PMA for 48 h; data are mean ± SEM of five independent experiments; growth obtained with vehicle was set as 100%. **c** Colony formation assay for HCT116, HT-29, and SW-837 cells treated with Roy-Bz; images correspond to a representative experiment of four; graphs represent mean ± SEM of four independent experiments. **d** Cell cycle arrest and **e** Annexin V-positive cells were determined for 24 h treatment with 1 μM Roy-Bz; data are mean ± SEM of four independent experiments. **f**, **g** Western blot analysis of (**f**) caspase-3 and (**g**) PARP cleavage, Bax, p53, Bcl-2, and survivin expression levels, for 24 h treatment with Roy-Bz in HCT116 cells. **h** Mitochondrial ROS generation (for 24 h) and ∆*ψ*_m_ dissipation (for 16 h) triggered by 0.5 μM Roy-Bz treatment in HCT116 cells; data are mean ± SEM of four independent experiments. **i** Mitochondrial cyt *c* release in HCT116 cells for 24 h treatment with Roy-Bz. **j** Evaluation of 3-day-old HCT116 spheroids diameter for 72 and 96 h treatment with Roy-Bz or vehicle. **k** CD44, ALDH2, MMP-9, and E-cadherin expression levels for 96 h treatment with 3.5 μM Roy-Bz. **l** Evaluation of spheroids diameter formed after 48 h treatment of seeded HCT116 cells with Roy-Bz or vehicle. In **j** and **l**, brightfield imaging of spheroids (scale bar = 50 μm, magnification = ×100); graphs represent mean ± SEM of four independent experiments. In **f**, **g**, **i** and **k** immunoblots represent one of three independent experiments; loading control of cytosolic (GAPDH) and mitochondrial (COX IV) fractions. In **c**, **d**, **e**, **h**, **j** and **l**, values significantly different from vehicle (*p < 0.05, **p < 0.01, ***p < 0.001), unpaired Student’s *t*-test

The effect of Roy-Bz on proliferation of human colorectal cancer cells was evaluated using a sulforhodamine B (SRB) assay (Fig. 3a, b). Treatment of HCT116, HT-29, and SW-837 cells (with similar PKCδ expression levels; Supplementary Fig. S1) with Roy-Bz for 48 h resulted in a dose-dependent inhibition of cell growth (IC_50_ values of 0.58 ± 0.05 μM for HCT116, 1.50 ± 0.06 μM for HT-29, and 1.08 ± 0.03 μM for SW-837; *n* = 5). The pronounced inhibitory effect of Roy-Bz on cell proliferation/viability of colorectal cancer cells was further demonstrated by assessing the colony-forming ability (Fig. 3c). In fact, even in the most resistant SW-837 cells, 0.3 μM Roy-Bz reduced the colony-forming ability by ~75% relative to vehicle.

Interestingly, while in HT-29 and SW-837 cells the Roy-Bz growth inhibitory effect was associated with G2/M-phase cell cycle arrest (Fig. 3d; particularly pronounced in HT-29) and apoptosis (Fig. 3e), in HCT116 cells the Roy-Bz-induced growth inhibition was only mediated by apoptosis. In fact, Roy-Bz had no effect on cell cycle progression (Fig. 3d; similar results were obtained for 8, 16, and 48 h treatment, data not shown) and markedly increased Annexin V-positive cells (Fig. 3e). Conversely, in HCT116 cells, PMA, with an IC_50_ value about 14-fold higher than that of Roy-Bz (7.83 ± 1.67 μM, *n* = 5; Fig. 3a), inhibited cell growth through induction of S-phase cell cycle arrest and apoptosis (Supplementary Fig. S2).

In HCT116 cells, the induction of apoptosis by Roy-Bz was further reinforced by the occurrence of caspase-3 and PARP cleavage, an increase in pro-apoptotic p53 and Bax levels, and a reduction in the levels of anti-apoptotic proteins Bcl-2 and survivin (Fig. 3f, g). The involvement of the mitochondrial pathway in Roy-Bz-induced apoptosis was also evidenced by the increase of mitochondrial reactive oxygen species (ROS) generation and ∆*ψ*_m_ dissipation (Fig. 3h), as well as the release of cytochrome *c* (cyt *c*) to cytosol (Fig. 3i).

In an attempt to explore the antitumor activity of Roy-Bz in a system that more closely resembles the in vivo features of the tumor microenvironment and is highly enriched in a small population of cancer stem cells (CSCs), a colonosphere culture model was generated from HCT116 cells. In fact, the spheroid-formation (colonosphere) assay is recognized as a valuable tool for assessment and expansion of stem cells in colon cancer^19^. In addition, according to several studies, colon CSCs can mainly be identified by the expression of cell biomarkers, such as CD44 and ALDH^20–23^. Actually, a positive correlation between the CD44 decrease and the therapeutic response of patients with colorectal cancer has been recently identified^22,23^. To test the effect of Roy-Bz in colonosphere growth, 3-day-old spheroids were treated with Roy-Bz for up to 96 h (Fig. 3j). A significant reduction in colonosphere diameter by Roy-Bz relative to vehicle was observed both at 72 h (at 3 and 3.5 μM) and 96 h (at 2–3.5 μM). Accordingly, 3.5 μM Roy-Bz markedly reduced the expression levels of the stemness markers CD44 and ALDH2 after 96 h treatment (Fig. 3k). Of note, a pronounced reduction of matrix metalloproteinase 9 (MMP-9) and increase of E-cadherin, prominent players in extracellular matrix homeostasis and metastasis^24^, were also observed under these treatment conditions (Fig. 3k). The effectiveness of Roy-Bz in colonosphere formation was also checked by evaluating the colonosphere diameter after 48 h treatment with Roy-Bz added at the seeding time of HCT116 cell suspension (Fig. 3l). The results showed a notable dose-dependent reduction in colonosphere formation ability by Roy-Bz, with an abolishment of colonosphere formation at 1 μM Roy-Bz.

Altogether, these results evidence a potent inhibitory activity of Roy-Bz in the growth of colon cancer cells, including in CSCs, suggesting a potential effect of the drug against cancer chemoresistance, dissemination, and recurrence.

### Roy-Bz pro-apoptotic and anti-migratory activity in HCT116 cancer cells is mediated by PKCδ-selective activation

**Fig. 4:**
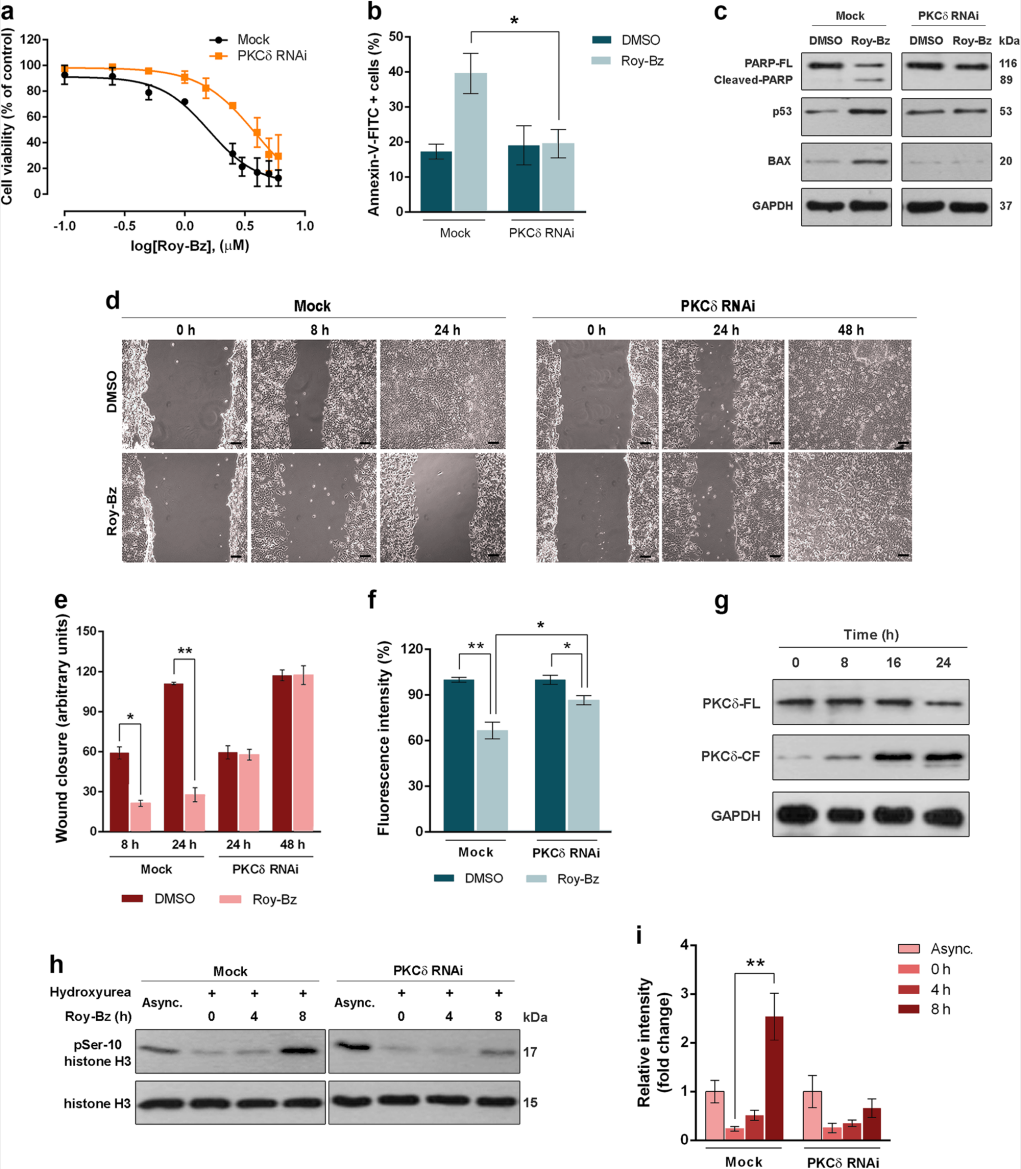
Pro-apoptotic and anti-migratory activity of Roy-Bz is mediated by selective activation of PKCδ in HCT116 cancer cells **a** Cell viability dose–response curves were determined by trypan blue assay for control (Mock) and PKCδ-knockdown (PKCδ RNAi) HCT116 cells treated with 0.1–6 μM Roy-Bz for 24 h; cell viability of vehicle was set as 100%. **b** Annexin V-positive cells were determined in control and PKCδ-knockdown HCT116 cells after 24 h treatment with 1 μM Roy-Bz or vehicle. **c** Western blot analysis of PARP cleavage, p53 and Bax expression levels for 16 h treatment with 1 μM Roy-Bz or vehicle in control and PKCδ-knockdown HCT116 cells; GAPDH (loading control). **d**,**e** Wound healing assay for control and PKCδ-knockdown HCT116 confluent cells treated with 0.25 µM Roy-Bz or vehicle, at different time-points; in **d**, scale bar = 50 µm and magnification = ×100; in **e**, quantification of wound closure in five randomly selected microscopic fields. **f** Chemotaxis migration assay for control and PKCδ-knockdown HCT116 cells for 24 h treatment with 0.25 µM Roy-Bz; migratory cells were quantified by fluorescence intensity, which was set as 100% for untreated cells. **g** Western blot analysis of full-length PKCδ (PKCδ-FL) and cleaved PKCδ fragment (PKCδ-CF) in HCT116 cells treated with 1 μM Roy-Bz for up to 24 h. **h**,**i** Levels of histone H3 phosphorylation on Ser-10 (pSer-10) in control and PKCδ-knockdown HCT116 cells treated with 1 μM Roy-Bz for up to 8 h; histone H3 (loading control); in **i**, quantification of pSer-10 levels normalized to histone H3, values of asynchronized cells were set as 1. In **c**, **g**, and **h**, immunoblots represent one of three independent experiments; in **a**,**b**,**e**,**f**, and **i** data are mean ± SEM of three independent experiments; values significantly different are indicated (*p < 0.05, **p < 0.01, ***p < 0.001), unpaired Student’s *t*-test

To evaluate the involvement of PKCδ in Roy-Bz-induced apoptosis, a PKCδ stable knockdown model was generated upon transfection of the metastatic colon cancer HCT116 cells with a PKCδ RNAi plasmid. As control, we used HCT116 cells transfected with non-target control RNAi. PKCδ silencing was confirmed by Western blot (Supplementary Fig. S3). The impact of 0.1–6 μM Roy-Bz on the viability of PKCδ-depleted and control HCT116 cells was thereafter evaluated by a trypan blue assay. As shown in Fig. 4a, silencing PKCδ decreased Roy-Bz-induced cell death compared to control HCT116 cells. Accordingly, the depletion of PKCδ in HCT116 cells abolished Roy-Bz-induced Annexin V-positive cells (Fig. 4b), PARP cleavage, and increase of Bax and p53 expression levels (Fig. 4c).

The impact of Roy-Bz on MMP-9 and E-cadherin expression levels in colonosphere cultures led us to further investigate the effect of Roy-Bz on the migration of HCT116 cells and its dependence on PKCδ. Using a wound healing assay, we found that Roy-Bz caused a pronounced inhibition of migration. Notably, the inhibitory effect of Roy-Bz was not observed in HCT116 cells subject to PKCδ-knockdown. It is important to note that at the concentration of Roy-Bz used in these assays (0.25 μM), there were no significant effects on cell viability (Fig. 4d, e). These results were also recapitulated using a chemotaxis cell migration assay, in which the anti-migratory effect of Roy-Bz observed in control HCT116 cells was significantly prevented by RNAi silencing of PKCδ expression (Fig. 4f).

It has been previously established in certain cellular models that PKCδ undergoes a proteolytic cleavage by caspases that generates a catalytically active fragment (PKCδ-CF)^6,7^. Interestingly, we found that in HCT116 cells, Roy-Bz caused a time-dependent increase in PKCδ-CF levels, without a visible interference in total PKCδ expression levels (Fig. 4g).

Since it was previously reported that activation of PKCδ is a required factor for histone H3 phosphorylation on Ser-10 (pSer-10 histone H3), which is a crucial event in apoptosis^25^, we next assessed the effect of Roy-Bz on pSer-10 histone H3 levels in HCT116 cells. To this end, HCT116 cells were synchronized in G1-phase by hydroxyurea, in order to exclude mitotic pSer-10 histone H3, followed by treatment with 1 μM Roy-Bz for up to 8 h. We observed that 1 μM Roy-Bz caused a pronounced increase of pSer-10 histone H3 levels that was noticeable at 8 h. This effect was abolished by PKCδ-knockdown (Fig. 4h, i), again reinforcing the concept that Roy-Bz is a potent inducer of apoptotic cancer cell death via activation of PKCδ.

Altogether, these results showed that Roy-Bz is a potent pro-apoptotic and anti-migratory agent in human colon cancer cells through PKCδ-selective activation.

### Roy-Bz is non-genotoxic in human cancer and normal cells and has in vivo PKCδ-dependent antitumor activity with no apparent toxic side effects

**Fig. 5:**
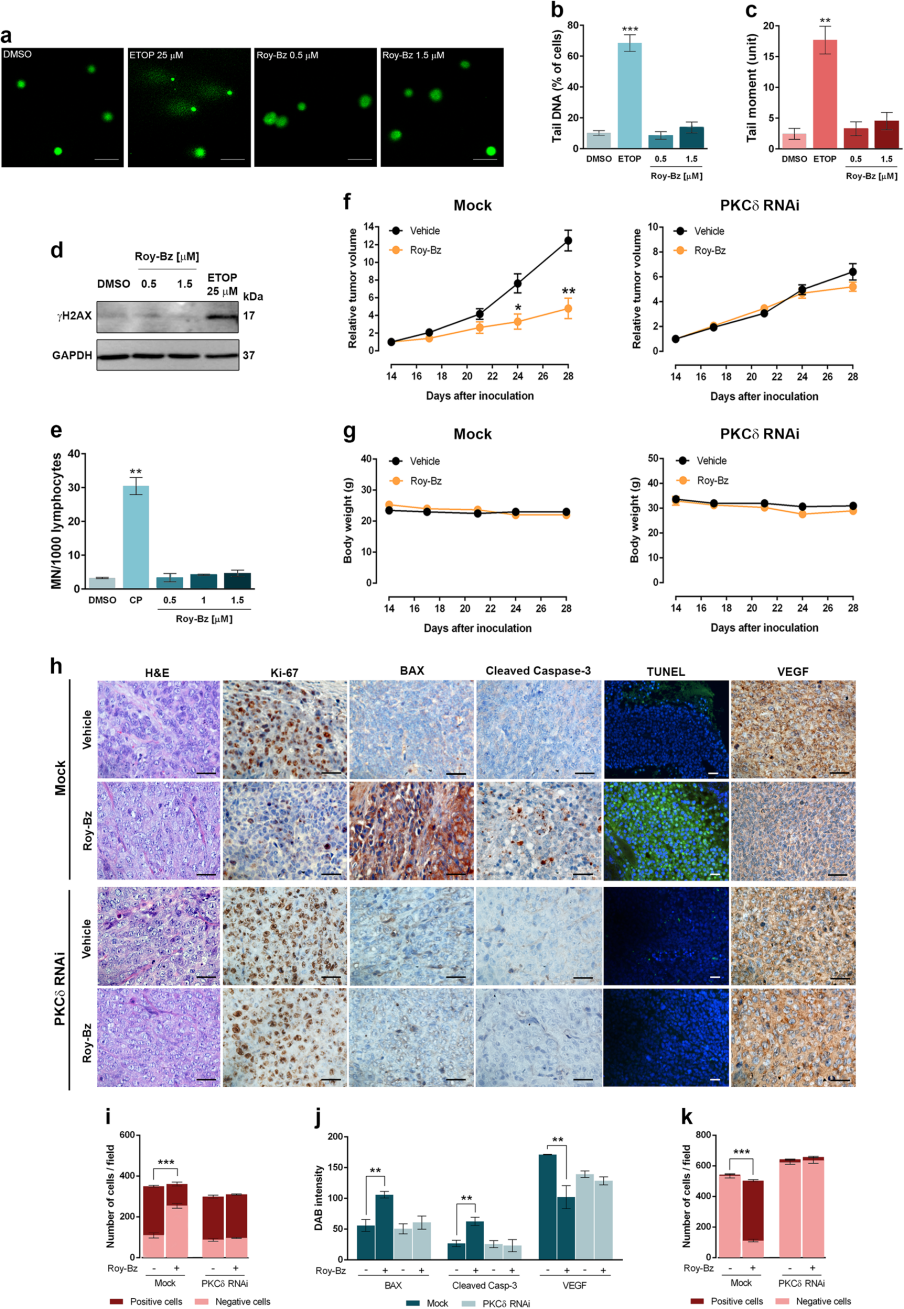
Roy-Bz is non-genotoxic in human cancer and normal cells and has PKCδ-dependent antitumor activity in human xenograft mouse models **a**–**d** Analysis of DNA damage was performed for 48 h treatment with ETOP, Roy-Bz, or vehicle; in **a**–**c**, comet assay in HCT116 cells; in **a**, scale bar = 20 μm; magnification = ×200; in **b** and **c**, one hundred cells were analyzed in each group; in **d**, western blot analysis of γH2AX levels; immunoblots are representative of three independent experiments; GAPDH (loading control). **e** Genotoxicity of Roy-Bz evaluated by cytokinesis-block micronucleus (MN) assay for 72 h treatment, in human lymphocyte cells; 5 μg/mL CP (positive control); the number of MN per 1000 binucleated lymphocytes was recorded. **f** Growth curves for relative tumor volume of BALB/c nude mice carrying control (Mock) and PKCδ-knockdown (PKCδ RNAi) HCT116 xenografts treated with vehicle or 10 mg/kg Roy-Bz; data are mean ± SEM of tumor volume fold change to the start of treatment, values significantly different from vehicle (*p < 0.05, **p < 0.01), unpaired Student’s *t*-test. **g** BALB/c nude mice body weight during vehicle or Roy-Bz treatment; no significant differences between vehicle-treated and Roy-Bz-treated mice: *p* > 0.05, unpaired Student’s *t*-test. **h** Representative images of Ki-67, BAX, cleaved caspase-3, DNA fragmentation (TUNEL) and VEGF detection in tumor tissues of control and PKCδ-knockdown HCT116 xenografts treated with Roy-Bz or vehicle, collected at the end of treatment (scale bar = 5 μm; magnification = ×400, ×200 for TUNEL); hematoxylin and eosin (H&E). **i–k** Quantification of immunohistochemistry of HCT116 xenograft tumor tissues of Mock and PKCδ RNAi treated with Roy-Bz or vehicle; in **i**, quantification of the number of positive and negative Ki-67 cells; in **j**, BAX, cleaved caspase-3 and VEGF staining quantification by evaluation of 3,3′-diaminobenzidine (DAB) intensity; in **k**, quantification of the number of positive and negative TUNEL cells. In **b**, **c**, **e**, **i**, **j**, and **k**, data are mean ± SEM of three independent experiments, values significantly different from vehicle (**p < 0.01, ***p < 0.001), unpaired Student’s *t*-test

The genotoxicity of Roy-Bz in human cancer cells was evaluated by checking comet-positive cells and histone H2AX phosphorylation on Serine-139 (γH2AX) as markers of DNA damage (single/double-strand breaks). Our results showed that, conversely to 25 μM etoposide (ETOP; positive control), 48 h treatment with Roy-Bz failed to induce DNA damage in HCT116 cells, as demonstrated by the negligible number of cells with more than 5% of DNA in tail (Fig. 5a, b), by the low tail moment (Fig. 5a, c), as well as by the absence of increased γH2AX levels compared to vehicle (Fig. 5d). Additionally, Roy-Bz failed to increase the number of micronuclei in peripheral lymphocytes of normal individuals, compared to vehicle (Fig. 5e).

We next examined potential primary toxicity signs induced by Roy-Bz treatment in vivo. To this end, Wistar rats were treated with 10 mg/kg Roy-Bz, vehicle, or saline solution (control) by intraperitoneal injection, twice a week during two weeks, followed by analysis of organs relative weight, biochemical, and hematological data (Table 1). No differences in the relative weight of liver, kidneys, heart, and spleen were observed among the three groups. Regarding biochemical data, despite the slight increase in uric acid and total proteins, compared to control groups, caused by Roy-Bz, the values of both parameters are in accordance with reference database^26,27^. These results indicated no apparent liver or kidney toxicity. Additionally, no differences in hematological data were observed between the three groups. Overall, no apparent toxic side effects were observed for Roy-Bz on the tissues most commonly affected by conventional chemotherapeutics.

**Table 1:** Toxicity studies of Roy-Bz in Wistar rats

	Saline	Vehicle	Treated
*Body weight and relative tissue weight (trophism)*			
BW (g)	340.30 ± 13.45	361.80 ± 0.01	308.25 ± 10.51
Heart/BW (g/kg)	3.03 ± 0.03	3.01 ± 0.09	3.33 ± 0.21
Liver/BW (g/kg)	38.71 ± 2,36	38.52 ± 0.89	42.06 ± 0.98
Kidney/BW (g/kg)	7.08 ± 0.40	6.81 ± 0.27	8.07 ± 0.31
Spleen (g/kg)	2.10 ± 0.20	2.15 ± 0.12	2.93 ± 0.14
*Biochemical data*			
Blood glucose(mg/dL)	188.00 ± 7.51	207.20 ± 7.00	155.00 ± 22.67
Urea (mg/dL)	20.67 ± 0.77	18.34 ± 0.24*	17.95 ± 0.59
Uric acid (mg/dL)	1.07 ± 0.07	0.82 ± 0.10	2.08 ± 0.45^#^
Creatinine (mg/dL)	0.30 ± 0.00	0.30 ± 0.01	0.29 ± 0.01
Total proteins (g/dL)	5.50 ± 0.00	5.35 ± 0.17	6.30 ± 0.11^#^
Albumin (g/dL)	3.03 ± 0.03	2.90 ± 0.05	3.13 ± 0.10
ALT (U/L)	36.00 ± 2.89	30.75 ± 3.30	45.75 ± 10.29
AST (U/L)	95.33 ± 25.36	56.25 ± 4.60	125.67 ± 41.79
Total chol (mg/dL)	44.33 ± 1.76	52.40 ± 3.54	67.00 ± 6.10
Triglycerides (mg/dL)	107.00 ± 16.29	161.40 ± 17.55	137.00 ± 0.07
*Hematological data*			
RBC count (×10^6^/µL)	7.10 ± 0.15	7.41 ± 0.28	7.84 ± 0.30
HGB (g/dL)	13.97 ± 0.18	13.95 ± 0.46	13.48 ± 0.54
HCT (%)	40.50 ± 0.82	42.28 ± 2.03	43.33 ± 1.89
WBC counts (×10^3^/µL)	1.93 ± 0.68	2.15 ± 0.88	1.55 ± 0.49
PLT counts (×10^3^/µL)	811.00 ± 29.61	793.80 ± 28.35	913.25 ± 81.96
RET counts (%)	2.80 ± 0.12	3.56 ± 0.23	4.65 ± 0.33

Data were analyzed for saline, vehicle and 10 mg/kg Roy-Bz (treated) rat groups, after four intraperitoneal administrations (twice a week). Results are mean ± SEM of four independent experiments

*ALT* alanine aminotransferase, *AST* aspartateaminotransferase, *BW* body weight, *CK* creatine kinase, *HCT* hematocrit, *HGB* hemoglobin concentration, *PCT* plateletcrit, *PLT* platelet, *RBC* red blood cell count, *RET* reticulocytes, *WBC* white blood cells

*p  <  0.05 (comparison between saline and vehicle groups; control groups)

^#^*p*   < 0.05 (comparison between Roy-Bz and control groups), and between vehicle and treated groups)

The in vivo antitumor potential of Roy-Bz was thereafter evaluated using human tumor xenograft mouse models of control and PKCδ-knockdown HCT116 cells, following the same administration procedure conducted in the toxicological studies. A significant inhibition in the growth of control HCT116 tumors was observed after administration of Roy-Bz (10  mg/kg) compared to vehicle (Fig. 5f, left panel). Notably, the antitumor activity of Roy-Bz was lost when mice where inoculated with PKCδ-depleted HCT116 cells (Fig. 5f, right panel), further reinforcing the concept that the antitumor activity of Roy-Bz was mediated by PKCδ. Of note, no significant body weight loss or morbidity signs were observed in Roy-Bz-treated mice compared to vehicle (Fig. 5g).

Lastly, proliferation, apoptosis, and angiogenesis markers were checked in tumor samples from control and PKCδ-knockdown HCT116 xenografts obtained at the end of in vivo antitumor assays (Fig. 5h–k). In PKCδ-expressing tumor samples, Roy-Bz reduced proliferation (decrease in Ki-67-positive staining) and stimulated apoptosis (increase in Bax expression, caspase-3 cleavage, and DNA fragmentation as demonstrated by TUNEL-positive staining), when compared to vehicle. The expression levels of vascular endothelial growth factor (VEGF) were also analyzed as a readout of angiogenesis. Data showed a marked reduction in VEGF expression in samples from tumors treated with Roy-Bz relative to vehicle (Fig. 5h, j). On the other hand, no apparent differences in these markers were observed between Roy-Bz and vehicle in PKCδ-knockdown tumor samples (Fig. 5h–k).

Altogether, these results demonstrated that Roy-Bz has strong in vivo PKCδ-dependent antitumor activity, through inhibition of proliferation and angiogenesis, and stimulation of apoptosis, with no apparent toxic side effects.

## Discussion

Despite the critical role of PKCs in numerous human diseases, the understanding of the functions of specific isozymes in a given disease remains under intense controversy due to the lack of PKC isozyme-selective modulators^1,2^. Herein, the compound Roy-Bz was identified as a potent and selective activator of PKCδ that, like phorbol esters, has the C1-domain as binding site. Additionally, it was shown that Roy-Bz has potent antitumor properties against colon cancer. Accordingly, it strongly inhibited the proliferation of colon cancer cells by triggering a PKCδ-dependent mitochondrial apoptotic pathway involving caspase-3 activation. The selectivity of Roy-Bz towards PKCδ in human colon cancer cells is further reinforced by the observation of the specific translocation of PKCδ, without any noticeable effect on the relocalization of other phorbol ester responsive PKC isozymes. Activation of PKCδ was also associated with the generation of a constitutive active PKCδ catalytic fragment, an effect observed in other models^6,7^, and the phosphorylation of histone H3 on Ser-10, a PKCδ-dependent event crucial in chromatin condensation during apoptosis^25^.

Despite the positive role of PKCδ in migration and invasiveness in some cellular models^28,29^, it was also disclosed that PKCδ suppressed the migration and the secretion of MMP-9 in highly motile breast cancer cells^30,31^. Supporting this anti-migratory activity of PKCδ, we found that Roy-Bz inhibited the migration of colon cancer cells in a PKCδ-dependent manner, with a reduction of MMP-9 and up-regulation of E-cadherin expression levels in colonospheres.

Recent studies have also demonstrated the relevant role of PKC isozymes in controlling cellular signaling of CSCs, which are critical in drug resistance, metastasis, and relapse of cancer^32^. However, further studies are required since scarce data are available on this subject. Our results support a negative regulation of stemness of colon cancer cells by PKCδ. Additionally, they indicate that Roy-Bz may also target drug-resistant CSCs, preventing tumor dissemination and recurrence, as evidenced by the pronounced reduction of colonosphere growth and formation, and by the depletion of the CD44 stemness marker. Interestingly, CD44 is a known downstream target of the Wnt/β-catenin pathway^33,34^, which is intricately involved in the growth and maintenance of colonospheres^35^. Accordingly, our results are in line with the recently reported inhibition of the Wnt/β-catenin pathway by PKCδ to suppress proliferation of colon cancer cells^15^.

Notably, a PKCδ-dependent antitumor effect of Roy-Bz, associated with anti-proliferative, pro-apoptotic, and anti-angiogenic activities, was demonstrated in xenograft mouse models. The great potential of Roy-Bz as an anticancer agent in colon cancer treatment was reinforced by the absence of genotoxicity in normal and tumor cells, as well as the lack of in vivo apparent toxic side effects.

PKCδ has been generally assumed as an anti-proliferative and pro-apoptotic kinase, and subsequently as a crucial death mediator of chemotherapeutic agents and radiotherapy^2,6,7,36^. Our experimental results with Roy-Bz in colon cancer cells are consistent with the notion that PKCδ preferentially acts as a tumor suppressor in intestinal carcinogenesis^11–15^. In fact, numerous studies have shown that PKCδ inhibits cell proliferation, as well as anchorage-dependent and anchorage-independent growth, and in addition it enhances differentiation of colon cancer cells^11–15^. PKCδ has also been implicated in growth inhibition and apoptosis of several other cancer types, including prostate and glioma^37,38^. However, anti-apoptotic and pro-survival roles of PKCδ have also been reported in other cancer types, particularly in breast cancer^39,40^, suggesting notable differences depending on the cellular context. It has been also reported that the tumor promoting properties of PKCδ after PMA treatment may result from the loss of PKC (down-regulation) due to its chronic application, rather than to its acute activation^41^. Interestingly, as observed with the PKC activator prostratin^42^, Roy-Bz did not significantly affect the PKCδ stability, what may explain the tumor suppression rather than tumor promoting activity of PKCδ associated with Roy-Bz.

In conclusion, in this work Roy-Bz was identified as the first small-molecule PKCδ-selective activator, with encouraging clinical application in colon cancer therapy. Roy-Bz opens the way to a new era on PKC biology and pharmacology. In particular, the elucidation of the structural requirements underlying its selectivity for PKCδ will be crucial to the structure-based design of other PKC isozyme-selective agents. In turn, these new agents will help in the elucidation of the specific functions of PKC isozymes in human diseases. Altogether, Roy-Bz will contribute to the redefinition of PKC isozymes as feasible therapeutic targets in precision medicine.

## Material and methods

### Compounds

7*α*-acetoxy-6*β*-benzoyloxy-12-*O*-benzoylroyleanone (Roy-Bz; Fig. 1a) was obtained by semi-synthesis from the natural diterpenoid 7*α*-acetoxy-6*β*-hydroxyroyleanone, isolated from a Lamiaceae family plant, as described in ref. ^43^. PMA and ARA were purchased from Enzo Life Science (Grupo Taper SA, Sintra, Portugal). ETOP, hydroxyurea, and cyclophosphamide (CP) were obtained from Sigma-Aldrich (Sintra, Portugal). All compounds were dissolved in dimethyl sulfoxide (DMSO; Sigma-Aldrich), except hydroxyurea and CP that were dissolved in water.

### Construction of the pESC-*LEU*-*Δ*C1-PKCδ plasmid

The wild-type (wt) *PRKCD* gene was amplified from the original clone kindly provided by Dr. Nigel Goode (The Royal Veterinary College, Hawkshead Lane, Hertfordshire, UK) using the gene-specific primers (Xho1 PKCδ F and NheI PKCδ R). After verifying the fragment by sequencing, it was cloned into the XhoI/NheI sites of pESC-*LEU* (Agilent Technologies) under the control of the *GAL1* promoter. According to manufacturer’s instructions, open reading frame (ORF) cloned in this vector will be expressed as a tagged protein with an N-terminal Myc-tag. Positive clones (pESC-*LEU*-PKCδ) were further confirmed by sequencing and maintained in *Escherchia coli* strain NovaBlue cells. The deletion of C1-domain in the wt *PRKCD* gene (Fig. 1c) was carried out by standard protocol of overlap primer extension using polymerase chain reaction (PCR)^44^ with the help of two outer gene-specific primers (Xho1 PKCδ F and NheI PKCδ R, Supplementary Table S1) and two inner primers (PKCδ C1-domain F and R, Supplementary Table S1). First, the region upstream and downstream to the C1-domain was amplified using outer and inner primer pair followed by overlapping PCR to obtain the final product lacking C1-domain. The final product was digested with XhoI and NheI and ligated with similarly digested pESC-*LEU*. Positive clones were sequenced in their entirety to confirm the desired modification at the corresponding position and maintained in *E. coli* Novablue cells.

### Yeast-based screening assay

*Saccharomyces cerevisiae* cells expressing mammalian PKCα, PKCβΙ, PKCδ, PKCε, or PKCζ were previously reported^16^. The constructed pESC-*LEU*-*Δ*C1-PKCδ plasmid was used to transform *S. cerevisiae* as described^16^. Yeast transformed with the empty vector was used as control. Cells were grown in galactose selective medium in the presence of PMA/ARA (positive controls), Roy-Bz, or vehicle (0.1% DMSO), for 42 h. Cell growth was analyzed by colony-forming unit counts^16^.

### Yeast cell cycle and cell death analysis

For yeast cell cycle analysis, 1 × 10^7^ cells were incubated with PMA, Roy-Bz, or vehicle, for 42 h. Cells were fixed and stained with 10 μM Sytox Green (Invitrogen, Alfagene, Carcavelos, Portugal) followed by flow cytometry analysis^16^. Propidium iodide (PI; Sigma-Aldrich) and TUNEL staining, using In Situ Cell Death Detection Kit Fluorescein (Roche Diagnostics, Sigma-Aldrich), were used to monitor necrosis and apoptosis, respectively^16^. Approximately 500 cells were counted in five random microscope fields using an Eclipse E400 fluorescence microscope (Nikon).

### In vitro PKC assay

The non-radioactive PKC kinase activity kit (ADI-EKS-420A, Enzo Life Sciences) and purified recombinant human PKC proteins, cPKCs (mix of PKCα, β, and γ), PKCδ, PKCε, and PKCζ (Millipore, VWR, Carnaxide, Portugal), were used. Briefly, 10 ng PKC was incubated with PMA/ARA, Roy-Bz, or vehicle for 1 h, and then transferred to a 96-well plate pre-coated with a peptide pseudosubstrate. A phosphospecific substrate antibody that recognizes the phosphorylated form of the substrate was added and detected using a peroxidase-conjugated antibody. The degree of PKC activation was directly proportional to the amount of phosphorylated substrate determined by measuring the OD_450_ (BioTeck Synergy HT Spectrophotometer). EC_50_ values were calculated considering the maximal response achieved with the positive control (constitutively activated PKC).

### Molecular docking

The crystallographic structure of PKCδ with PDB code 1PTR having PRB as co-crystallized ligand^45^ was used to test the binding mode of Roy-Bz. First, the co-crystallized PRB molecule was re-docked to test the efficiency of the docking procedure in reproducing the experimental binding pose. The binding pose was reproduced with autodock 4.2 with a RMSD of 0.47 Å. The exact same conformation was reproduced with the molecule being slightly less inserted into the protein. All autodock docking parameters were produced with Autodock Tools and were kept at the default. The docking box was centered on the crystallographic position of the PRB molecule having 42, 62, and 36 grid points in *x*, *y*, and *z* with a grid spacing of 0.375.

### Human cancer cell lines and culture conditions

Human colon (HCT116 and HT-29) and colorectal (SW-837) adenocarcinoma cell lines were purchased from ATCC. All cancer cells were cultured in RPMI-1640 medium with ultraglutamine (Lonza, VWR, Carnaxide, Portugal), and supplemented with 10% fetal bovine serum (FBS; Gibco, Alfagene, Carcavelos, Portugal). Cells were maintained at 37 °C in a humidified atmosphere of 5% CO_2_.

### Cell proliferation and viability assay

Cell proliferation was determined using the SRB assay^46^. Briefly, cells were seeded in 96-well plates at 5.0 × 10^3^ (for HCT116 and HT-29) and 7.5 × 10^3^ (for SW-837) cells/well and 24 h later treated with PMA, Roy-Bz, or vehicle for 48 h. IC_50_ (concentration that causes 50% of growth inhibition) values were determined from the concentration–response curves. The percentage of viable cells was determined using the Trypan Blue Assay. Briefly, HCT116 cells were seeded in 24-well plates at 6 × 10^4^ cells/well for 24 h, followed by treatment with Roy-Bz for 24 h. Cells were then harvested and resuspended in 0.4% Trypan Blue (Sigma-Aldrich), and the number of viable/dead cells were counted using a Leica light optical microscope (Wetzlar). Vehicle (0.25% DMSO) was included as a control.

### Colony formation assay

Cells were seeded in six-well plates at 5.0 × 10^2^ (for HCT116 and SW-837) and 1.0 × 10^3^ (for HT-29) cells/well, followed by incubation with Roy-Bz or vehicle for 11 days. Colonies were fixed using 10% methanol and 10% acetic acid for 10 min and stained with 0.5% crystal violet (Sigma-Aldrich) in 1:1 methanol/H_2_O for 15 min. Colonies containing more than 20 cells were counted.

### Generation of colon cancer spheroids (colonosphere assay)

HCT116 cells were resuspended in serum-free stem cell culture media consisting of Dulbecco’s modified Eagle medium supplemented with 10 ng/mL bFGF and 20 ng/mL EGF (Bio-techne, Citomed Lda, Lisboa, Portugal), 1× B27 (Life Technologies), and 5 μg/mL insulin (Sigma-Aldrich). Cells were plated in 24-well ultra-low attachment plates (Corning Inc.; one spheroid/well) at 1 × 10^3^ cells/well. Treatments with Roy-Bz or vehicle were performed 3 days after spheroid formation for up to 96 h, or at the seeding time for 48 h. Spheroid formation was monitored using an inverted Nikon TE 2000-U microscope at ×100 magnification with a DXM1200F digital camera and using Nikon ACT-1 software. Spheroid diameters were quantified using the ImageJ software.

### Cell cycle and apoptosis

Cells were seeded in six-well plates at 1.5 × 10^5^ (for HCT116 and HT-29) or 2.25 × 10^5^ (for SW-837) cells/well for 24 h, followed by treatment with Roy-Bz or vehicle for 24 h. For cell cycle analysis, cells were stained with PI and analyzed by flow cytometry^46^. For apoptosis, cells were analyzed by flow cytometry using the Annexin V-FITC Apoptosis Detection Kit I (BD Biosciences, Enzifarma, Porto, Portugal) according to the manufacturer’s instructions^46^.

### ROS generation and mitochondrial membrane potential (∆*ψ*_m_)

HCT116 cells were seeded in six-well plates, at 1.5 × 10^5^ cells/well for 24 h. For mitochondrial ROS generation, cells were treated with Roy-Bz or vehicle for 24 h followed by incubation with 3 μM MitoSOX (Invitrogen) for 30 min at 37 °C. For ∆*ψ*_m_ dissipation, cells were treated with Roy-Bz or vehicle for 16 h, followed by incubation with 1 nM DiOC_6_^3^ (Invitrogen) for 30 min at 37 °C^47^. Cells were analyzed by flow cytometry.

### Western blot

HCT116 cells were seeded in six-well plates, at 1.5 × 10^5^ cells/well and 24 h later treated with different compounds for the indicated times. Protein extracts from total, mitochondrial, and cytosolic fractions of cancer cells were obtained and analyzed by Western blot as described^46,47^. Antibodies are described in Supplementary Table S2.

### Generation of stable PKCδ-knockdown cell lines

HCT116 cells were seeded in 24-well plates at 6 × 10^4^ cells/well for 24 h. For the generation of stable cell lines, cells were co-transfected with 1 μg of control pSuper plasmid (control; mock) or 900 ng of pSuperPKCδ.RNAi [Addgene plasmid #10819;^48^] and 100 ng of pSUPERpuro plasmids, and 2 μg/ml Lipofectamine 2000 (Invitrogen) for 72 h. Stable transfectants were selected with 3 μg/ml puromycin (Sigma-Aldrich) for 4 weeks. Clones were screened for PKCδ expression silencing by Western blot using an anti-PKCδ antibody (Santa Cruz Biotechnology, Frilabo, Porto, Portugal) (Supplementary Fig. S3).

### Migration assay

Cell migration was analyzed using PKCδ-knockdown and control HCT116 cells, using both the wound healing assay and the QCM 24-Well Fluorimetric Chemotaxis Cell Migration Kit (8 µm; Merck Millipore)^49,50^. In the wound healing assay, confluent HCT116 cells with a wound were treated with Roy-Bz or vehicle for up to 48 h. Cells were photographed using the Moticam 5.0MP camera with a Motic’s AE2000 inverted microscope (×100 magnification). Wound closure was calculated by subtracting the ‘wound’ area (measured using Image J software) at the indicated time-point of treatment from the ‘wound’ area at the starting point. For the Chemotaxis Cell Migration Kit, 5 × 10^5^ cells/ml of HCT116 cells were treated with Roy-Bz or vehicle, for 24 h. Cells that migrated through the 8 μm pore membranes were eluted, lysed, and stained with a green-fluorescence dye. The number of migrated cells was proportional to the fluorescence signal measured using the Bio-Tek Synergy HT plate reader at 480/520 nm (ex/em).

### Immunofluorescence and confocal microscopy

HCT116 cells were transfected with pEGFP-N1-PKCα, pEGFP-N1-PKCδ, or pEGFP-N1-PKCε using Lipofectamine 3000. Forty-eight hours later, cells were treated with Roy-Bz, PMA (positive control), or vehicle for 1 h. Cellular localization of GFP-fused PKCs was analyzed using a Nikon TE2000-U microscope, as described^18^.

### Comet assay

HCT116 cells were seeded in six-well plates at 1.5 × 10^5^ cells/well for 24 h, followed by treatment with ETOP, Roy-Bz, or vehicle, for 48 h. To evaluate DNA damage, a neutral comet assay was performed as described^50^. Tail DNA quantification considers the percentage of cells with more than 5% of DNA in the tail (assessed by Open Comet/ImageJ); tail moment corresponds to the product of the tail length and the % of DNA in the tail. Cells were photographed using a Nikon DS-5Mc camera and a Nikon Eclipse E400 fluorescence microscope, and images processed using a Nikon ACT-2U software (Izasa).

### Micronucleus assay

Genotoxicity was analyzed by the cytokinesis-blocked micronucleus assay in lymphocytes^49,50^. Briefly, fresh peripheral blood samples were collected from healthy volunteers and were treated for 44 h with 5 μg/mL CP (positive control), Roy-Bz, or vehicle, followed by treatment with 3 μg/mL cytochalasin B (Sigma-Aldrich) for 28 h. Lymphocytes were isolated, fixed, and stained with Wright stain (Sigma-Aldrich). For each sample, 1000 binucleated lymphocytes were blindly scored using a Leica light optical microscope (Wetzlar). The number of micronuclei per 1000 binucleated lymphocytes was recorded.

### In vivo antitumor and toxicity assays

Animal experiments were conducted according to the EU Directive 2010/63/EU and the National Authorities. Female BALB/c nude mice (~14-week-old) and male Wistar rats (~12-week-old) were purchased from Charles-River Laboratories and housed under pathogen-free conditions in individual ventilated cages. For the toxicity assays, Wistar rats were treated with 10 mg/kg Roy-Bz, vehicle, or saline solution (control) by intraperitoneal injection, twice a week during 2 weeks. Samples of blood and organs (heart, liver, kidney, and spleen) were then collected for toxicological analysis. Each group was composed of four animals. Xenograft tumor assays were performed with HCT116 cells stably transfected with control pSUPER plasmid or pSuperPKCδ-RNAi. Briefly, 1 × 10^6^ cells (in PBS) were inoculated subcutaneously in the dorsal flank of mice. Tumor dimensions were assessed by caliper measurement and their volumes were calculated [tumor volume = (*L* × *W*^2^)/2; *L* and *W* represent the longest and shortest axis of the tumor, respectively]. Treatment was started when tumors reached a volume of ~100 mm^3^. Mice were treated twice a week for 2 weeks with 10 mg/kg Roy-Bz or vehicle by intraperitoneal injection. Tumor volumes and body weights were monitored twice a week until the end of the treatment. Animals were sacrificed by cervical dislocation at the end of the study, when tumors reached ~1500 mm^3^, or the animals present any signs of morbidity. Each group was composed of six animals.

### Immunohistochemistry

Tumor tissues were fixed in 10% formalin, embedded in paraffin, sectioned at 4 µm, and stained with hematoxylin and eosin (H&E) or antibodies as described^49,50^. Antibodies are described in Supplementary Table S2. Immunostaining was carried out using the UltraVision Quanto Detection System HRP 3,3′-diaminobenzidine (DAB) kit (Lab Vision Thermo Scientific, Grupo Taper SA, Sintra, Portugal), according to the manufacturer’s instructions. Images were obtained using the Moticam 5.0MP camera with Motic’s AE2000 inverted microscope (×400 magnification). Evaluation of DAB intensity and quantification of marked cells were performed using Image J software. TUNEL assay was performed using the In situ Cell Death Detection Kit Fluorescein (Roche), according to the manufacturer’s instructions. Tissues were counterstained with DAPI (0.1 μg/ml). Images were obtained using an Eclipse E400 fluorescence microscope (Nikon) with ×200 magnification, with Digital Sight camera system (Nikon DS-5Mc) and software Nikon ACT-2U.

### Flow cytometric data acquisition and analysis

The Accuri^TM^ C6 flow cytometer and the CellQuest software (BD Biosciences) were used. The FlowJo software was used to identify and quantify cell cycle phases.

### Statistical analysis

Data were statistically analyzed using the GraphPad Prism. Differences between means were tested for significance using the Student’s *t*-test and two-way analysis of variance (ANOVA).

## Electronic supplementary material

Supplementary Figure S1

Supplementary Figure S2

Supplementary Figure S3

Supplementary Table S1

Supplementary Table S2
