# Supplementary material for: Discovery of a small-molecule protein kinase Cδ-selective activator with promising application in colon cancer therapy
Source: Cell Death Dis. 2018 Jan 18;9(2):23. doi: 10.1038/s41419-017-0154-9 (PMC5833815; doi:10.1038/s41419-017-0154-9)
Supplement: Supplementary file 1 — Supplementary Figure S1 [file 41419_2017_154_MOESM1_ESM.docx]

**Supplementary Figure S1. PKCδ expression levels in colorectal cancer cells**

**
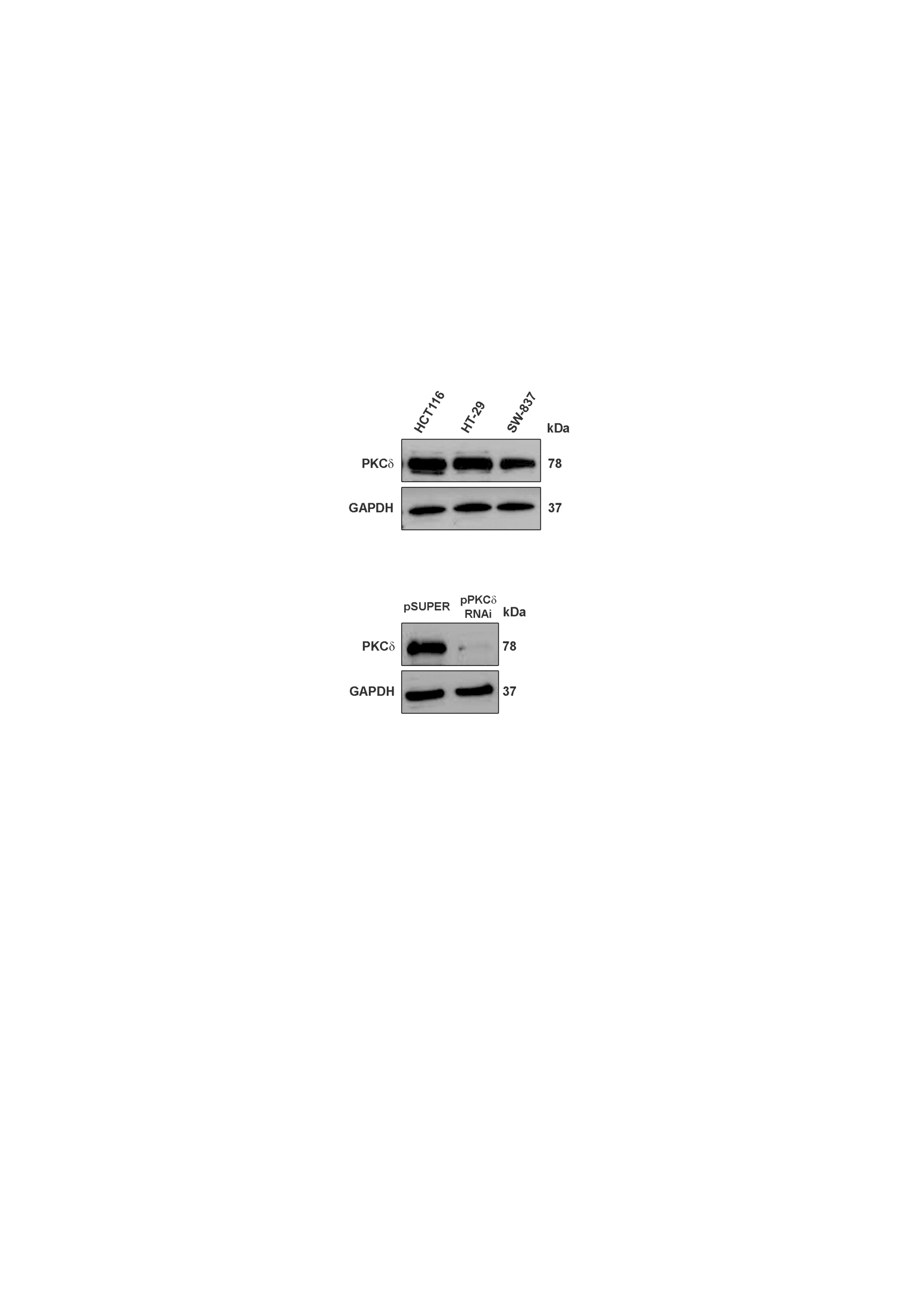
**

Western blot analysis of PKCδ expression levels assessed in HCT116, HT-29 and SW-837 cells after 48 h incubation. Immunoblots represent one of three independent experiments; GAPDH was used as loading control.
