# Supplementary material for: Discovery of a small-molecule protein kinase Cδ-selective activator with promising application in colon cancer therapy
Source: Cell Death Dis. 2018 Jan 18;9(2):23. doi: 10.1038/s41419-017-0154-9 (PMC5833815; doi:10.1038/s41419-017-0154-9)
Supplement: Supplementary file 2 — Supplementary Figure S2 [file 41419_2017_154_MOESM2_ESM.docx]

**Supplementary Figure S2. Effect of PMA on cell cycle and apoptosis of HCT116 colon cancer cells**

**
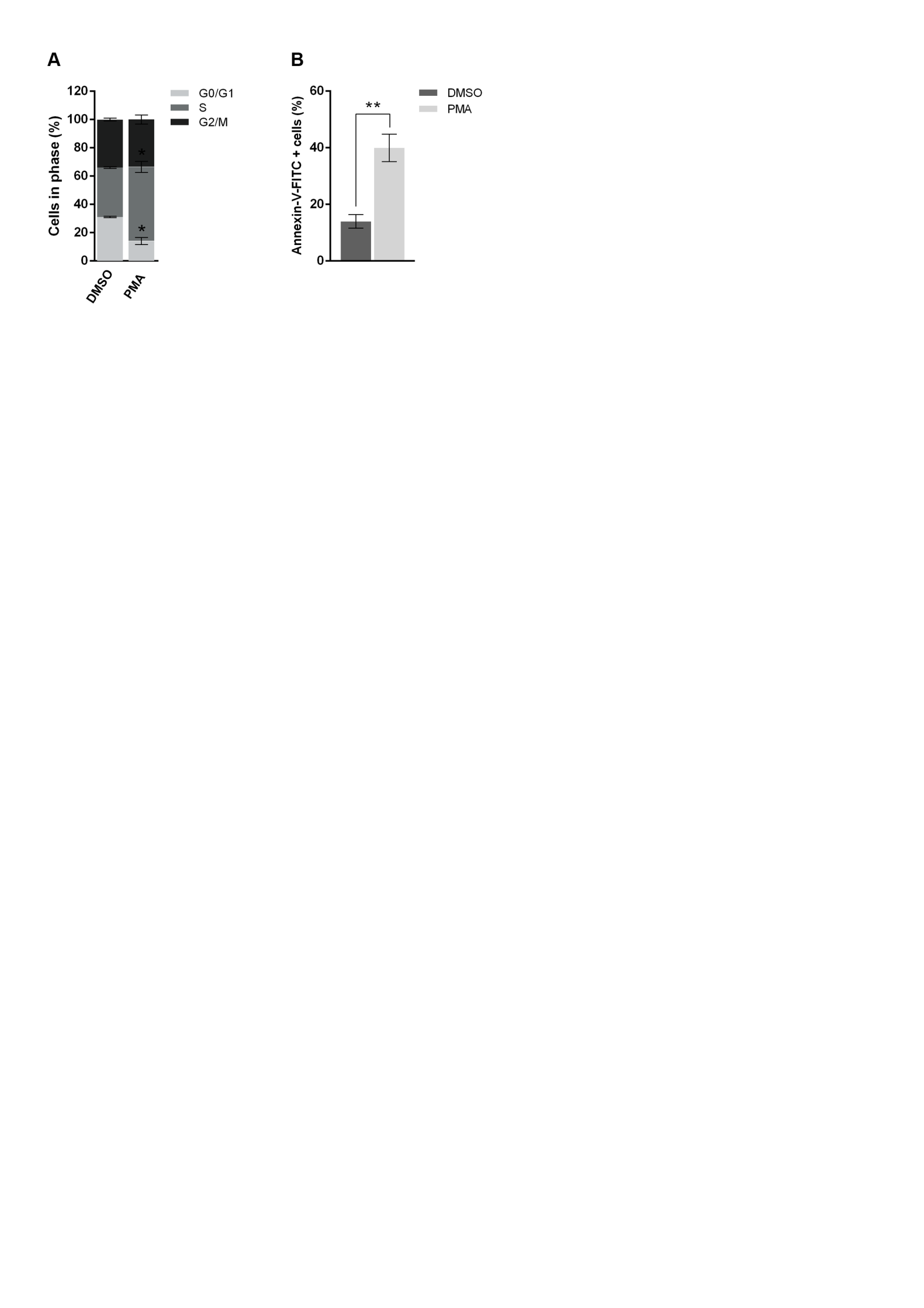
**

(**a**) Cell cycle (**b**) and apoptosis were analyzed after 24 h treatment with 8 μM PMA or vehicle in HCT116 cells. Data are mean ± SEM of four independent experiments; values significantly different from vehicle: (**p*<0.05, ***p*<0.01), unpaired Student’s *t*-test.
