# Supplementary material for: Discovery of a small-molecule protein kinase Cδ-selective activator with promising application in colon cancer therapy
Source: Cell Death Dis. 2018 Jan 18;9(2):23. doi: 10.1038/s41419-017-0154-9 (PMC5833815; doi:10.1038/s41419-017-0154-9)
Supplement: Supplementary file 3 — Supplementary Figure S3 [file 41419_2017_154_MOESM3_ESM.docx]

**Supplementary Figure S3. PKCδ-knockdown in HCT116 colon cancer cells**


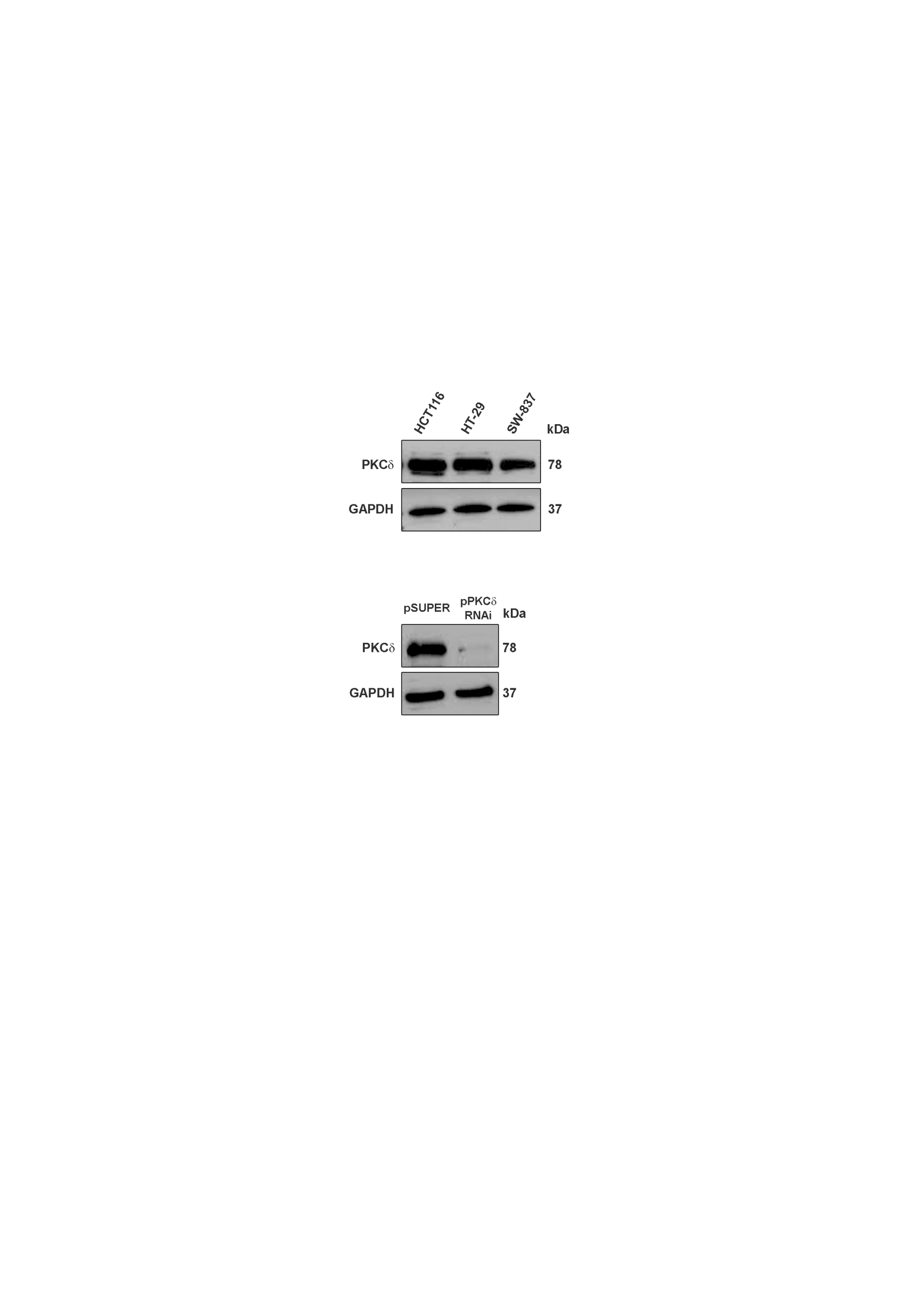


Efficiency of PKCδ-knockdown was assessed by Western blot analysis after 24 h post-transfection with control plasmid (pSUPER) or pSuperPKCδ.RNAi. Immunoblots represent one of three independent experiments; GAPDH was used as loading control.
