# Supplementary material for: Discovery of a small-molecule protein kinase Cδ-selective activator with promising application in colon cancer therapy
Source: Cell Death Dis. 2018 Jan 18;9(2):23. doi: 10.1038/s41419-017-0154-9 (PMC5833815; doi:10.1038/s41419-017-0154-9)
Supplement: Supplementary file 4 — Supplementary Table S1 [file 41419_2017_154_MOESM4_ESM.docx]

**Supplementary Table S1.** **Primer sequence used in PCR reactions**

| Primers | Sequence |
| --- | --- |
| XhoI PKCδ F (outer primer) | 5’-CCGCTCGAGATGGCCCCGTTCCTGCGCATC-3’ |
| NheI PKCδ R (outer primer) | 5’- CTAGCTAGCCTATTCCAGGAATTGCTC-3’ |
| PKCδ C1 del F (inner primer) | 5’-AACATCGACATGCCTCAAAAGCTCTTAGCTG-3’ |
| PKCδ C1 del R (inner primer) | 5’- CAGCTAAGAGCTTTTGAGGCATGTCGATGTT-3’ |

F: forward primer; R: reverse primer
