# Supplementary material for: Discovery of a small-molecule protein kinase Cδ-selective activator with promising application in colon cancer therapy
Source: Cell Death Dis. 2018 Jan 18;9(2):23. doi: 10.1038/s41419-017-0154-9 (PMC5833815; doi:10.1038/s41419-017-0154-9)
Supplement: Supplementary file 5 — Supplementary Table S2 [file 41419_2017_154_MOESM5_ESM.docx]

**Supplementary Table S2. List of antibodies used in the work**

| Antigen | Final Dilution | Supplier |
| --- | --- | --- |
| Primary antibodies | | |
| Caspase-3 (#9662)  (rabbit polyclonal) | 1:300  (Western blot) | Cell Signaling Technology |
| Phospho-Histone H3 (Ser10) (#9701)  (rabbit polyclonal) | 1:100  (Western blot) |  |
| Histone H3 (#9715)  (rabbit polyclonal) | 1:1000  (Western blot) |  |
| PARP (C2-10)  (mouse monoclonal) | 1:1000  (Western blot) | Santa Cruz Biotechnology |
| p53 (DO-1)  (mouse monoclonal) | 1:5000  (Western blot) |  |
| BAX (2D2)  (mouse monoclonal) | 1:100  (Western blot) |  |
| Bcl-2 (C-2)  (mouse monoclonal) | 1:200  (Western blot) |  |
| PKCδ (G-9)  (mouse monoclonal) | 1:300  (Western blot) |  |
| Cytochrome *c* (A-8)  (mouse monoclonal) | 1:50  (Western blot) |  |
| HCAM (CD44) (F-4)  (mouse monoclonal) | 1:200  (Western blot) |  |
| COX IV (F-8)  (mouse monoclonal) | 1:500  (Western blot) |  |
| GAPDH (6C5)  (mouse monoclonal) | 1:10000  (Western blot) |  |
| Survivin (ab76424)  (rabbit monoclonal) | 1:10000  (Western blot) | Abcam |
| ALDH2 (ab108306)  (rabbit monoclonal) | 1:1000  (Western blot) |  |
| MMP-9 (ab38898)  (rabbit polyclonal) | 1:200  (Western blot) |  |
| Histone γH2AX (phospho-Ser139)  (rabbit monoclonal) | 1:50  (Western blot) |  |
| Myc  (rabbit polyclonal) | 1:500  (immunofluorescence) | Millipore |
| Caspase-3 (AB3623)  (rabbit polyclonal) | 1:100  (immunohistochemistry) |  |
| Ki-67 (SP6)  (rabbit monoclonal) | 1:200  (immunohistochemistry) | Pierce Thermo Scientific |
| BAX (6A7)  (mouse monoclonal) | 1:200  (immunohistochemistry) |  |
| VEGF (VG1)  (mouse monoclonal) | 1:200  (immunohistochemistry) |  |
|  | **Secondary antibodies** |  |
| Alexa Fluor 488-coupled antibody | 1:2000  (immunofluorescence) | Invitrogen |
| Anti-mouse horseradish-peroxidase (HRP)-conjugated | 1:5000  (Western blot) | Santa Cruz Biotechnology |
| Anti-rabbit horseradish-peroxidase (HRP)-conjugated | 1:5000  (Western blot) |  |
